# Single-use negative-pressure wound therapy *versus* conventional dressings for closed surgical incisions: systematic literature review and meta-analysis

**DOI:** 10.1093/bjsopen/zraa003

**Published:** 2020-12-18

**Authors:** C Saunders, L M Nherera, A Horner, P Trueman

**Affiliations:** 1 Global Clinical Affairs, Smith+Nephew, Hull, UK; 2 Health Economics and Market Access, Smith+Nephew, Hull, UK

## Abstract

**Background:**

Surgical-site complications (SSCs) remain a significant cause of morbidity and mortality, particularly in high-risk patients. The aim of this study was to determine whether prophylactic use of a specific single-use negative-pressure wound therapy (sNPWT) device reduced the incidence of SSCs after closed surgical incisions compared with conventional dressings.

**Methods:**

A systematic literature review was performed using MEDLINE, Embase and the Cochrane Library to identify articles published from January 2011 to August 2018. RCTs and observational studies comparing PICO™ sNPWT with conventional dressings, with at least 10 patients in each treatment arm, were included. Meta-analyses were performed to determine odds ratios (ORs) or mean differences (MDs), as appropriate. PRISMA guidelines were followed. The primary outcome was surgical-site infection (SSI). Secondary outcomes were other SSCs and hospital efficiencies. Risk of bias was assessed.

**Results:**

Of 6197 citations screened, 29 studies enrolling 5614 patients were included in the review; all studies included patients with risk factors for SSCs. sNPWT reduced the number of SSIs (OR 0.37, 95 per cent c.i. 0.28 to 0.50; number needed to treat (NNT) 20). sNPWT reduced the odds of wound dehiscence (OR 0.70, 0.53 to 0.92; NNT 26), seroma (OR 0.23, 0.11 to 0.45; NNT 13) and necrosis (OR 0.11, 0.03 to 0.39; NNT 12). Mean length of hospital stay was shorter in patients who underwent sNPWT (MD −1.75, 95 per cent c.i. −2.69 to −0.81).

**Conclusion:**

Use of the sNPWT device in patients with risk factors reduced the incidence of SSCs and the mean length of hospital stay.

## Teaser

A systematic literature review and meta-analysis was performed for a specific single-use negative-pressure wound therapy (sNPWT) device to determine whether there were improvements in outcomes compared with conventional dressings. Odds of surgical-site infection, dehiscence, seroma and necrosis were reduced by 63, 30, 77 and 89 per cent respectively. There was a 1.75-day reduction in length of hospital stay in favour of sNPWT.

Reduces SSI in patients with risk factors

## Resumen

**Antecedentes:**

Las complicaciones del sitio quirúrgico (*surgical site complications*, SSCs) siguen siendo una causa importante de morbilidad y mortalidad, especialmente en pacientes de alto riesgo. El objetivo de este estudio fue determinar si el uso profiláctico de un dispositivo específico de tratamiento de heridas con presión negativa de un solo uso (*single-use negative pressure wound therapy*, sNPWT) reducía la incidencia de SSCs después de incisiones quirúrgicas cerradas, en comparación con los apósitos convencionales.

**Métodos:**

Se llevó a cabo una revisión sistemática de la literatura (*systematic literature review*, SLR) utilizando Medline, Embase y la Biblioteca Cochrane para identificar los artículos publicados entre enero de 2011 y agosto de 2018. Se seleccionaron ensayos clínicos aleatorizados y controlados (*randomised controlled trials*, RCTs) y estudios observacionales que comparaban PICO™ sNPWT con apósitos convencionales con al menos 10 pacientes incluidos en cada brazo de tratamiento. Se realizaron metaanálisis para determinar la razón de oportunidades (*odds ratio*, OR) o la diferencia de medias (*mean difference*, MD), según correspondiere. Se siguieron las recomendaciones PRISMA. El criterio de valoración principal fueron las infecciones del sitio quirúrgico (*surgical site infections*, SSIs). Los resultados secundarios eran otros CSS y las eficiencias hospitalarias. Se evaluó el riesgo de sesgo.

**Resultados:**

De 6.197 referencias examinadas, se seleccionaron 29 estudios con 5.614 pacientes, todos los cuales presentaban factores de riesgo de SSCs. El sNPWT redujo las SSI (OR: 0,39; i.c. del 95%: 0,29-0,52; número necesario a tratar (NNT): 20). El sNPWT redujo las probabilidades de dehiscencia de la herida (OR: 0,70; i.c. del 95%: 0,53-0,92; NNT: 26), seroma (OR: 0,23; i.c. del 95%: 0,11-0,45; NNT: 13) y necrosis (OR: 0,11; i.c. del 95%: 0,03-0,39; NNT: 12). La duración media de la estancia hospitalaria fue más corta en los pacientes que se sometieron a sNPWT (diferencia media: -1,75; i.c. del 95%: -2,69 a -0,81).

**Conclusión:**

El uso del dispositivo sNPWT en pacientes con factores de riesgo redujo la incidencia de SSCs y la duración media de la estancia hospitalaria.

## Introduction

Postoperative surgical-site complications (SSCs) represent a significant burden to healthcare systems. SSCs such as surgical-site infection (SSI), dehiscence, seroma and haematoma can delay the healing process, cause abnormal wound healing, and result in the formation of hypertrophic and keloid scars^1^. These complications can result in increased length of hospital stay, higher rates of readmission, and compromised health outcomes, thereby escalating costs associated with a patient’s episode of care. This emphasizes the importance of preventing SSCs to ensure delivery of optimal and cost-effective patient care pathways by healthcare professionals and policy-makers.

The incidence of SSI after surgery is an important outcome measure for the success of a closed surgical-incision wound management treatment pathway. For this complication in particular, concerns over the increasing prevalence of antibiotic-resistant strains of bacteria necessitates consideration of alternative therapies. There are many factors that can influence wound healing and the potential for infection, including patient-related (high BMI, smoking status, pre-existing co-morbidities and high ASA grade) and procedural-related factors (type of surgery, prolonged duration of surgery, use of synthetic implants and increased complexity of surgery)^2^. Clear guidance, however, on what constitutes a high-risk patient is currently lacking.

Optimizing wound outcomes is complex and multifactorial, particularly within high-risk patient populations, but single-use negative-pressure wound therapy (sNPWT) devices have emerged as an important technological innovation in wound management. Differences in product specifications between the sNPWT devices offered by different companies, particularly for pressure settings, therapy duration and exudate management, may lead to variations in the clinical benefit offered by each^3^. PICO™ (Smith+Nephew, Hull, UK) is a canister-free sNPWT system consisting of a sterile pump and multilayered adhesive dressings. The device is used in place of conventional postsurgical wound dressings in closed surgical incision wounds with low to moderate exudate level. The dressing can be left in place for up to 7 days, and can be used in both hospital and community settings. Of particular relevance to closed surgical incisions, the system contains a unique AIRLOCK™ Technology layer (Smith+Nephew, Hull, UK) that delivers consistent negative pressure across the whole dressing to ensure treatment is delivered to a wider zone beyond the wound itself^4^.

The aim of this study was to determine whether use of the PICO™ system could reduce the incidence of SSCs in comparison with conventional dressings. In addition, the effect on hospital efficiencies was investigated. The review was performed as part of a health technology assessment of the PICO™ NPWT system for the National Institute for Health and Care Excellence (NICE) in England and Wales.

## Methods

This review was written in accordance with PRISMA guidelines^5^. Although the review was not preregistered, the protocol and outcomes to be studied were agreed in advance with NICE before conducting the review.

### Search strategy

MEDLINE, Embase and the Cochrane Library were searched for relevant articles published between January 2011 to August 2018. Search terms used included ‘negative pressure wound therapy’ OR NPWT OR PICO OR ‘topical negative pressure’. To increase the sensitivity of searches, search terms were intentionally left open and did not include words related to specific outcomes, patient populations or adverse events. Reference lists of included articles were hand-searched to identify any further potentially eligible studies that may have been missed by the database search strategy. Searches were restricted to English-language articles, but no other filters were applied. ClinicalTrials.gov and ISRCTN trial registers were checked to ensure that no additional studies were available.

### Study selection

Two experienced data reviewers screened potentially relevant articles independently by examining the titles and abstracts. All abstracts were assessed using the inclusion and exclusion criteria listed in *Table 1*, and studies were included if they fulfilled the criteria. If either reviewer deemed an article as potentially relevant, the article progressed to full-text screening. In case of disagreement, a third reviewer made the final decision after reading the full-text paper or conference abstract. Included studies compared outcomes following the use of PICO™ *versus* standard care for closed surgical incisions. The standard of care was defined as the use of standard non-NPWT dressings.

**Table 1:** Inclusion and exclusion criteria used to identify relevant studies for inclusion in the systematic literature review and meta-analysis

	Inclusion criteria	Exclusion criteria
Population	Patients of any age with closed surgical incisions. Patients with any risk factors for complications were also included	Patients with open surgical incisions or any non-surgical wound
Intervention	PICO™ (Smith+Nephew, Hull, UK) (single-use NPWT) applied after surgery on a closed surgical incision. Participants undergoing any type of operation were eligible, and both prophylactic and reactive use of PICO™ were included	Other forms of NPWT (not PICO™) were excluded
Comparator	Standard care (any non-NPWT dressing)	Non-standard care
Outcome	Surgical-site infections, dehiscence, oedema, seroma, haematoma, skin/fat necrosis, delayed healing, abnormal scarring, readmission rates, length of hospital stay, reoperation rates, number of dressing changes, time to heal	n.a.
Study design	RCTs or retrospective/prospective observational studies with at least 10 patients in each treatment arm	Case reports, case series, studies with fewer than 10 patients in each treatment arm, letters, commentaries, notes, reviews and editorials
Language restrictions	English	Not in English
Search dates	Studies published from 1 January 2011 to 1 August 2018	Studies published before 2011

NPWT, negative-pressure wound therapy; n.a., not applicable.

### Data extraction and quality assessment

Data were extracted from included studies by one reviewer using a predefined and standardized data extraction form, and checked for accuracy by a second reviewer. Extracted data included descriptions of study characteristics. Study design, location of the study, the number of patients involved and the type of surgery performed were recorded.

The primary outcome of interest for this systematic literature review was the number of patients who had an SSI with PICO™ compared with the standard of care. Secondary outcomes of interest were the number of patients who experienced dehiscence, oedema, seroma, haematoma, skin/fat necrosis, delayed healing or abnormal scarring after surgery. Readmission rates, reoperation rates, number of dressing changes, length of hospital stay and time to heal were recorded. Studies were also screened for reporting of potential device-related issues.

### Quality assessment

The reviewers assessed risk of bias for each study, recognizing the challenges of blinding participants to the sNPWT device. For RCTs, Centre for Reviews and Dissemination guidelines^6^ for the assessment of risk of bias in RCTs were followed. For observational studies, Critical Appraisal Skills Programme (CASP) guidelines were followed, using criteria that were adopted by NICE for their Medical Technologies Evaluation Programme^7^. Funnel plots were produced to determine potential risk of bias from the cumulative evidence.

### Statistical analysis

Meta-analyses were performed in Review Manager^®^ V5.3 (The Nordic Cochrane Centre, Copenhagen, Denmark). Heterogeneity of included studies was assessed using the *I*^2^ statistic. When *I*^2^ was less than 50 per cent (indicating no substantial heterogeneity), a fixed-effect model was used; when *I*^2^ was over 50 per cent, a random-effects model was used. For dichotomous outcomes, the Mantel–Haenszel method was used to combine separate statistics, and the odds ratio (OR) with 95 per cent c.i. was reported as the summary statistic. For continuous outcomes, the inverse variance method was used to combine statistics, and the mean difference (MD) was used and expressed using usual units (for example, days for length of stay). Relative risk was calculated and used in number needed to treat (NNT) calculations.

Sensitivity analyses were performed using alternative pooling methods (for example, Peto method *versus* Mantel–Haenszel method, applicable to dichotomous data). Further sensitivity analyses were the inclusion and exclusion of conference abstracts and using fixed-effect or random-effects models. *P*<0.050 denoted statistical significance.

## Results

**Fig. 1:**
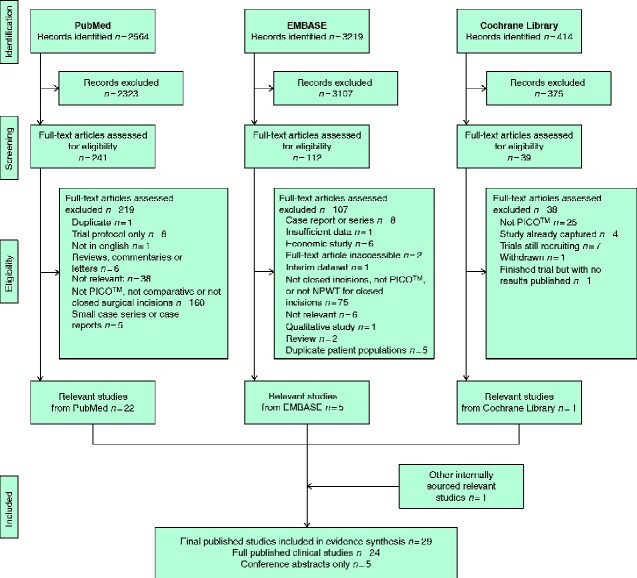
PRISMA diagram for the review NPWT, negative-pressure wound therapy.

A total of 2564 articles were identified from PubMed, 3219 from Embase, and 414 from the Cochrane Library. A PRISMA diagram of the number of studies at each stage of the process is shown in *Fig. 1*. After applying the inclusion and exclusion criteria, 29 studies^8–36^ were selected. Of these studies, 11 were RCTs, 13 were observational studies, and five were available as conference abstracts.

### Study characteristics

Key characteristics for each included study are shown in *Table S1*. These studies represent patients from a wide geographical distribution, including five studies from the UK^14^^,^^15^^,^^17^^,^^19^^,^^20^, two from Ireland^11^^,^^24^, nine from mainland Europe^10^^,^^18^^,^^23^^,^^25–27^^,^^30^^,^^33^^,^^34^, one from the Nordic region^28^, six from the USA^8^^,^^16^^,^^21^^,^^22^^,^^31^^,^^36^, two from Australia^9^^,^^13^, two from Asia^29^^,^^32^, and one from Mexico^35^. One additional study was a multicentre study incorporating patients from the USA, France, the Netherlands and South Africa^12^. A range of surgical specialties were represented within the identified evidence, including orthopaedics, obstetrics, colorectal, breast, vascular and cardiothoracic surgery. In all studies, factors could be identified that placed the patient populations at higher risk of developing an SSC due to patient- or procedure-related factors.

### Risk of bias and confidence in the evidence

The overall quality of the included studies was deemed acceptable (*Tables 2* and *3*). For RCTs, the largest source of bias identified was the inability to ensure blinding of outcome assessors. An intention-to-treat analysis was performed in some, but not all, RCTs, potentially introducing another source of bias. Outcome reporting was considered to be complete for all studies, except for the study by Galiano *et al.*^12^, in which the results of scar quality were not included.

**Table 2:** Assessment of risk of bias for RCTs included in the analysis

	**Chaboyer *et al.*** ^9^	**Galiano *et al.*** ^12^	**Gillespie *et al.*** ^13^	**Hyldig *et al.*** ^18^	**Karlakki *et al.*** ^20^	**Nordmeyer *et al.*** ^23^	**O’Leary *et al.*** ^24^	**Svensson-Björk *et al.*** ^28^	**Tanaydin *et al.*** ^30^	**Uchino *et al.*** ^32^	**Witt-Majchrzak *et al.*** ^34^
Was the method used to generate random allocations adequate?	Yes	Yes	Yes	Yes	Yes	?	Yes	Yes	Yes	Yes	?
Was the allocation adequately concealed?	Yes	Yes	Yes	Yes	Yes	?	Yes	Yes	Yes	Yes	?
Were the groups similar at the outset of the study?	?	Yes	Yes	Yes	?	?	Yes	Yes	Yes	Yes	?
Were care providers, participants and assessors blinded to treatment allocation?	n.a.	n.a.	n.a.	n.a.	n.a.	n.a.	n.a.	n.a.	n.a.	n.a.	n.a.
Were any drop-outs balanced between groups?	Yes	Yes	Yes	Yes	Yes	Yes	Yes	Yes	Yes	Yes	Yes
Have all outcomes measured by the authors being reported, or is there evidence to suggest otherwise?	Yes	?	Yes	Yes	Yes	Yes	Yes	Yes	Yes	Yes	Yes
Was an ITT analysis included? If so, were appropriate methods used to account for missing data?	No	No	?	Yes	Yes	No	?	No	No	Yes	No

Quality criteria were taken from Centre for Reviews and Dissemination guidelines for the assessment of risk of bias in RCTs^6^. ?, Partial or unclear; n.a., not applicable; ITT, intention to treat.

**Table 3:** Assessment of risk of bias for observational studies included in the analysis

	Adogwa *et al.*^8^	Dingemans *et al.*^10^	Fleming *et al.*^11^	Gupta *et al.*^36^	Hester *et al.*^15^	Hickson *et al.*^16^	Holt and Murphy^17^	Matsumoto and Parekh^22^	Pellino *et al.*^26^	Pellino *et al.*^25^	Selvaggi *et al.*^27^	Tan *et al.*^29^	van der Valk *et al.*^33^
Was the cohort recruited in an acceptable way?	Yes	Yes	Yes	Yes	Yes	Yes	Yes	Yes	Yes	Yes	Yes	?	?
Was the exposure accurately measured to minimize bias?	Yes	Yes	Yes	Yes	Yes	Yes	No	Yes	Yes	Yes	Yes	?	Yes
Was the outcome accurately assessed to minimize bias?	Yes	Yes	Yes	Yes	Yes	Yes	Yes	Yes	Yes	Yes	Yes	Yes	No
Have the authors identified all important confounding factors?	Yes	Yes	Yes	?	Yes	Yes	Yes	Yes	Yes	Yes	Yes	Yes	Yes
Have the authors taken account of the confounding factors in the design and/or analysis?	Yes	Yes	Yes	Yes	?	Yes	Yes	Yes	Yes	Yes	Yes	?	Yes
Was the follow-up of patients complete?	Yes	Yes	Yes	Yes	Yes	Yes	Yes	Yes	Yes	Yes	Yes	Yes	?
Are the results precise (for example, were confidence intervals and *P* values provided)?	No	Yes	Yes	Yes	?	No	No	Yes	Yes	Yes	Yes	Yes	?

Criteria were adapted from Critical Appraisal Skills Programme guidelines^7^. ?, Partial or unclear.

### Meta-analysis of outcomes of surgical-site complications

**Fig. 2:**
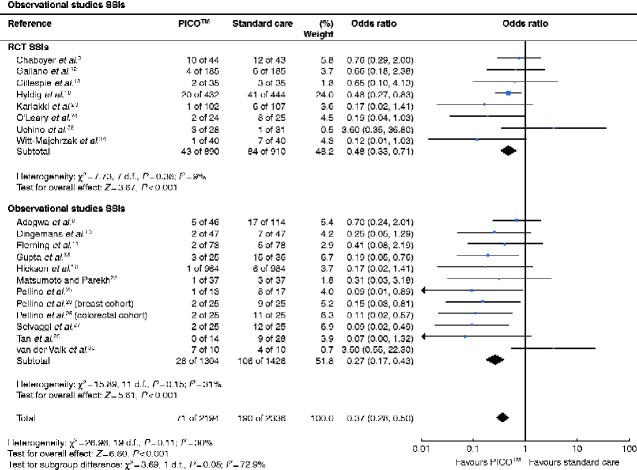
Forest plots of surgical-site infection in patients treated with PICO™ *versus* standard care A Mantel–Haenszel fixed-effect model was used for meta-analysis. Odds ratios are shown with 95 per cent confidence intervals.

An overview of the results from meta-analyses of SSCs is shown in *Table 4*. Overall, the odds of SSI were reduced by 63 per cent with the use of the PICO™ (OR 0.37, 95 per cent c.i. 0.28 to 0.50), with the corresponding forest plot shown in *Fig. 2*. This corresponded to a NNT of 20 for this outcome. When the results from RCTs and observational studies were considered in isolation, use of the PICO™ resulted in a reduction of 52 per cent (OR 0.48, 0.33 to 0.71) and 73 per cent (OR 0.27, 0.17 to 0.43) respectively. Sensitivity analyses showed that this statistically significant reduction was maintained when a random-effects model was used instead of a fixed-effect model, and also when data from conference abstracts were included (data not shown). When studies were segmented based on the type of surgery and subanalyses were performed, the significant reduction in SSI was maintained for orthopaedic (OR 0.43, 0.21 to 0.86; NNT 15), breast (OR 0.36, 0.14 to 0.97; NNT 23), vascular (OR 0.22, 0.05 to 0.87; NNT 9) and obstetric (OR 0.49, 0.31 to 0.78; NNT 54) surgery. For colorectal surgery, statistical significance was not reached (OR 0.43, 0.09 to 2.05).

**Table 4:** Results of meta-analyses performed for surgical-site complications

Outcome or subgroup	No. of studies	No. of participants	Statistical method	*I* ^2^ statistic (%)		Effect estimate	*P*
SSI (all operations)^8–13^^,^^16^^,^^18^^,^^20^^,^^22^^,^^24–27^^,^^29^^,^^32–34^^,^^36^	19	4530	OR (M–H, fixed effect, 95% c.i.)	30	0.37 (0.28, 0.50)		<0.001
Orthopaedic surgery SSI^8^^,^^10^^,^^13^^,^^20^^,^^22^	5	607	OR (M–H, fixed effect, 95% c.i.)	0	0.43 (0.21, 0.86)		0.02
Colorectal surgery SSI^25^^,^^27^^,^^32^^,^^33^^,^^36^	5	220	OR (M–H, random effects, 95% c.i.)	72	0.43 (0.09, 2.05)		0.29
Obstetric surgery SSI^9^^,^^16^^,^^18^	3	2911	OR (M–H, fixed effect, 95% c.i.)	0	0.49 (0.31, 0.78)		0.003
Breast surgery SSI^12^^,^^26^	2	420	OR (M–H, fixed effect, 95% c.i.)	46	0.36 (0.14, 0.97)		0.04
Vascular surgery SSI^11^^,^^29^	2	193	OR (M–H, fixed effect, 95% c.i.)	11	0.22 (0.05, 0.87)		0.03
Cardiothoracic surgery SSI^34^	1	80	OR (M–H, fixed effect, 95% c.i.)	n.a.	0.12 (0.01, 1.03)		0.05
Mixed surgery SSI^24^	1	49	OR (M–H, fixed effect, 95% c.i.)	n.a.	0.19 (0.04, 1.03)		0.05
Dehiscence^8^^,^^11–13^^,^^17^^,^^18^^,^^30^^,^^33^^,^^34^	9	1790	OR (M–H, fixed effect, 95% c.i.)	0	0.70 (0.53, 0.92)		0.01
Seroma^11–13^^,^^25–27^	6	771	OR (M–H, fixed effect, 95% c.i.)	17	0.23 (0.11, 0.45)		<0.001
Haematoma^11–13^	3	591	OR (M–H, fixed effect, 95% c.i.)	0	1.02 (0.35, 2.97)		0.96
Time to healing^11^^,^^32^^,^^33^	3	259	MD (IV, random effects, 95% c.i.)	86	−17.91 (− 44.64, 8.81)		0.19
Delayed healing^12^^,^^17^^,^^20^	3	627	OR (M–H, fixed effect, 95% c.i.)	0	0.77 (0.51, 1.16)		0.21
Necrosis^12^^,^^34^	2	474	OR (M–H, fixed effect, 95% c.i.)	38	0.11 (0.03, 0.39)		<0.001
Abnormal scarring^34^	1	80	OR (M–H, fixed effect, 95% c.i.)	n.a.	0.38 (0.09, 1.60)		0.19
Length of stay^8^^,^^9^^,^^11^^,^^13^^,^^20^^,^^25–29^	10	948	MD (IV, random effects, 95% c.i.)	92	−1.75 (− 2.69, −0.81)		<0.001
Readmission rates^8^^,^^9^^,^^11–13^^,^^25^^,^^27^^,^^29^^,^^33^	9	966	OR (M–H, fixed effect, 95% c.i.)	0	0.82 (0.49, 1.38)		0.45
Reoperation rates^8^^,^^17^^,^^18^^,^^24^^,^^25^^,^^27^^,^^29^^,^^33^^,^^34^	9	1385	OR (M–H, fixed effect, 95% c.i.)	0	0.92 (0.54, 1.56)		0.75

Values in parentheses are 95 per cent confidence intervals. SSI, surgical-site infection; OR, odds ratio; M–H, Mantel–Haenszel; n.a., not applicable; MD, mean difference; IV, inverse variance.

When other SSCs were considered, there was a difference in favour of sNPWT for dehiscence (OR 0.70, 95 per cent c.i. 0.53 to 0.92; NNT 26). sNPWT also reduced seroma (OR 0.23, 0.11 to 0.45; NNT 13) and necrosis (OR 0.11, 0.03 to 0.39; NNT 12). Heterogeneity, as indicated by the *I*^2^ statistic, was considered not significant (less than 50 per cent) in all meta-analyses except from SSI for colorectal surgery (72 per cent), time to healing (86 per cent) and length of hospital stay (92 per cent).

All other SSCs analysed (haematoma, delayed healing, abnormal scarring and time to healing) demonstrated no difference between sNPWT and standard of care. Data on the number of dressing changes and oedema were insufficient to perform meta-analyses. When studies were screened for potential device-related adverse events, 3^9^^,^^20^^,^^34^ of the 29 studies described a tendency towards more blisters/serous vesicles in the sNPWT group. These were, however, reported as minor, and resolved without further complications. Two studies^10^^,^^33^ also reported problems in maintaining a vacuum in a small number of patients.

### Meta-analysis of hospital efficiency outcomes

As shown in *Table 4*, length of hospital stay was reduced in patients treated with PICO™ compared with that of patients treated with conventional dressings (MD −1.75, 95 per cent c.i. −2.69 to −0.81). Sensitivity analysis indicated that this difference was maintained regardless of whether a fixed-effect or random-effects model was used. No difference was found in readmission (OR 0.82, 0.49 to 1.38) or reoperation (OR 0.92, 0.54 to 1.56) rate.

### Publication bias

**Fig. 3:**
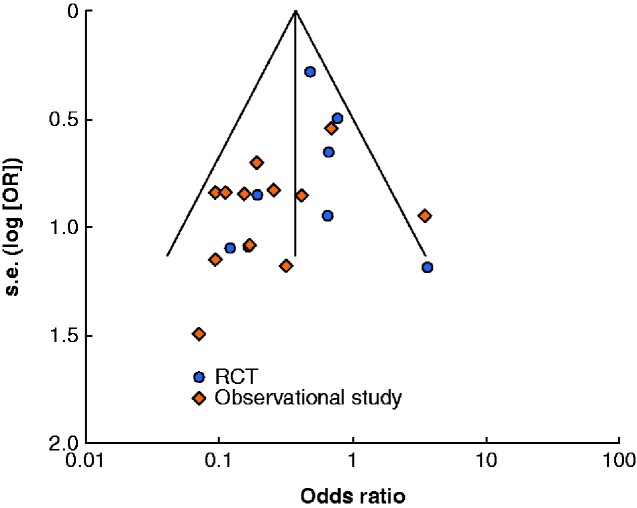
Funnel plot for surgical-site infection outcome to assess publication bias For each clinical study included in the meta-analysis, the reported odds ratio (OR) was plotted against the standard error (s.e.). The overall effect average is shown by the vertical line, and the diagonal lines represent the region in which 95 per cent of trials were expected to lie.

A funnel plot was produced to assess for publication bias in the 19 included clinical studies used in the comparison of SSI outcome (*Fig. 3*). The distribution of studies was approximately symmetrical, although RCTs were more predominant on the right side of the graph and observational studies more predominant on the left. All but one study lay within the region where 95 per cent of studies were predicted to be in the absence of bias and heterogeneity.

## Discussion

In this meta-analysis, sNPWT reduced several clinically important SSCs, including SSI, dehiscence, seroma and necrosis, compared with standard of care. For SSI specifically, this reduction was seen across a range of surgical specialties, including orthopaedics, breast surgery, vascular surgery and obstetrics. In addition, sNPWT reduced the length of hospital stay.

All studies included in the present analysis had patient populations with risk factors for SSCs, although the exact criteria used to define a patient as high risk differed between studies. This was likely due to lack of guidance on how to identify clearly patients at high risk of SSCs and, as a result, the patients within each study may have had differing risk profiles for developing complications. Thus, there may be clinical heterogeneity between studies, and this should be considered when extrapolating the results to local practice. It has been shown in subgroup analyses presented by Galiano and colleagues^12^ and Pellino *et al.*^26^ that PICO™ may have increasing benefit over the standard of care with increasing patient age and BMI. Thus, the absolute percentage reduction reported for complications such as SSI and wound dehiscence may differ, depending on the risk profile of an individual patient. It is likely to be the case that patients with more risk factors may see a greater reduction in the incidence of SSCs.

Subgroup analyses support the use of sNPWT to reduce SSIs for certain specialties. The variability of risk factors present within the patient cohorts of each study makes it difficult to estimate the size of the effect for each surgical procedure separately. However, SSCs are a common concern across different surgical specialties, and the mechanism of action of PICO™ on a surgical incision should be transferable between different procedures.

The bias assessment performed revealed some potential sources of bias that should be considered. In most cases it was not possible to blind the patient and treating clinician to treatment assignment, owing to the nature of the device. The use of a sham device could have been possible, and was considered by the authors of some studies^12^, but it would likely still be apparent which treatment a patient had been assigned to. An alternative way to overcome this could be to ensure an independent and blinded assessor for the reporting of wound outcomes specifically. The majority of the studies, however, did not specify whether this was the case. The lack of intention-to-treat analyses in some of the studies may have led to attrition bias. A further limitation of the data is that some of the studies included had small sample sizes.

The major strength of the clinical evidence presented in this review is the depth and breadth of the evidence base for the use of this sNPWT device after closed surgical incisions. Many of these studies were published within the last 24 months, allowing for timely comparison with the current standard of care in many cases. Observational studies were also included. Although these are inherently subject to bias, the observational nature of these studies can often provide high external validity. The consistency of the results between different studies, and the relatively low bias identified for the observational studies included, suggest that the inclusion of observational studies was unlikely to have had a significant impact on the overall conclusions.

The results of this analysis suggest that this technology warrants consideration by policy-makers and healthcare professionals to optimize postsurgical wound treatment pathways, to ensure that all patients have the best treatment while making effective use of scarce healthcare resources.

## Funding information

The authors are employees of Smith+Nephew.

## Supplementary Material

zraa003_Supplementary_Data

## Notes

Presented to the 29th Conference of the European Wound Management Association, Gothenburg, Sweden, June 2019
