# Supplementary material for: Single-use negative-pressure wound therapy versus conventional dressings for closed surgical incisions: systematic literature review and meta-analysis
Source: BJS Open. 2020 Dec 18;5(1):zraa003. doi: 10.1093/bjsopen/zraa003 (PMC7893467; doi:10.1093/bjsopen/zraa003)
Supplement: zraa003_Supplementary_Data [file zraa003_supplementary_data.zip › Title page.docx]

**Outcomes with PICO single-use negative pressure wound therapy compared to conventional dressings for closed surgical incisions: systematic literature review and meta-analysis**

Christopher Saunders, PhD MBChB*^1^

Leo M Nherera, MSc^2^

Alan Horner, PhD^1^

Paul Trueman, MA^2^

^1^Global Clinical Affairs, Smith & Nephew plc, Hull, United Kingdom

^2^Health Economics & Market Access, Smith & Nephew plc, Hull, United Kingdom

*Corresponding author/requests for reprints: Christopher Saunders, 101 Hessle Road, Hull, HU3 2BN, United Kingdom. Christopher.Saunders@smith-nephew.com, 01482673781

Sources of funding: Authors are employees of Smith & Nephew.

Manuscript category: Review

Previous communication to a society: 29^th^ Conference of the European Wound Management Association; Gothenburg, Sweden; EP065 (June 2019). A single-use negative pressure system reduces surgical site complications compared with conventional dressings in closed surgical incisions: a systematic literature review with meta-analysis.
