# Supplementary material for: Single-use negative-pressure wound therapy versus conventional dressings for closed surgical incisions: systematic literature review and meta-analysis
Source: BJS Open. 2020 Dec 18;5(1):zraa003. doi: 10.1093/bjsopen/zraa003 (PMC7893467; doi:10.1093/bjsopen/zraa003)
Supplement: zraa003_Supplementary_Data [file zraa003_supplementary_data.zip › zraa003_Table_S1.docx]

| **TABLE S1** **Characteristics of relevant studies identified in the systematic literature review** | | | | |
| --- | --- | --- | --- | --- |
| Study | Study design and location | Number of patients and surgery performed | Identified risk factors for surgical site complications | Outcomes of interest reported |
| Randomised controlled trials | | | | |
| Chaboyer *et al* 2014^9^ | Pilot, single centre RCT. Australia | 92 elective caesarean section patients | - Inclusion criterion of BMI ≥30kg/m^2^ | SSI, dehiscence, haematoma, seroma, length of hospital stay, readmission rate |
| Galiano *et al* 2018^12^ | Multi-centre open-label RCT. United States, France, South Africa, Netherlands | 200 bilateral reduction mammoplasty patients | - The majority of patients had a raised BMI and a large mass of tissue resected | SSI, dehiscence, seroma, delayed healing, skin/fat necrosis, haematoma, readmission rate |
| Gillespie *et al* 2015^13^ | Pilot, single centre, open-label RCT. Australia | 70 elective primary hip arthroplasty patients | - The majority of patients had an ASA score of ≥2 | SSI, dehiscence, haematoma, seroma, length of hospital stay, readmission rate |
| Hyldig *et al* 2018^18^ | Multi-centre RCT. Denmark | 876 elective and emergency caesarean section patients | - Inclusion criterion of BMI ≥30kg/m^2^ | SSI, dehiscence, reoperation rate |
| Karlakki *et al* 2016^20^ | Single centre, open-label RCT. United Kingdom | 220 patients undergoing elective hip and knee arthroplasty | - The majority of patients had a raised BMI and ASA score - The mean age of participants was >65 years old | SSI, delayed healing, length of hospital stay |
| Nordmeyer *et al* 2016^23^ | Single centre, open-label RCT. Germany | 20 spinal fracture patients who received internal fixation | - Use of internal fixation devices | Number of dressing changes |
| O’Leary *et al* 2017^24^ | Single centre, open-label RCT. Ireland | 50 laparotomy patients who received open abdominal surgery | - The majority of patients had a raised BMI and ASA score - Type of surgery | SSI, reoperation rate |
| Svensson-Björk *et al* 2018^28^ | Multi-centre, open label RCT. Sweden | 34 patients undergoing bilateral inguinal vascular surgery | - The mean age of participants was >65 years old - The majority of patients had co-morbidities | SSI, length of hospital stay |
| Tanaydin *et al* 2018^30^ | Single centre, open label RCT. Netherlands | 32 patients undergoing bilateral breast reduction mammoplasty | - The majority of patients had a large mass of tissue resected | Dehiscence |
| Uchino *et al* 2016^32^ | Single centre, open label RCT. Japan | 59 patients with ulcerative colitis undergoing elective ileostomy closure | - All patients had a raised ASA score - Inclusion criterion of patients with ulcerative colitis | SSI, time to healing |
| Witt-Majchrzak *et al* 2015^34^ | Single centre, open label RCT. Poland | 80 patients undergoing coronary artery bypass grafting surgery | - The majority of patients had a raised BMI and co-morbidities - Prolonged duration of surgery | SSI, dehiscence, necrosis, abnormal scarring, reoperation rates |
| Observational studies | | | | |
| Adogwa *el al* 2014^8^ | Retrospective, before-after, single-centre, observational study. United States | 160 patients undergoing thoracolumbar fusion for spinal deformity | - Use of screws and rod instrumentation devices - The majority of patients had a raised BMI and co-morbidities | SSI, dehiscence, length of hospital stay, 30-day readmission rate, reoperation rate |
| Dingemans *et al* 2018^10^ | Pilot before-after observational study. Netherlands | 60 patients with foot or ankle fractures | - Type of surgery | SSI |
| Fleming *et al* 2018^11^ | Retrospective, single-centre observational study. Ireland | 151 patients undergoing vascular surgery for groin wounds | - The mean age of participants was >65 years old | SSI, dehiscence, seroma, haematoma, length of hospital stay, readmission rate, time to healing |
| Gupta *et al* 2017^36^ | Retrospective, single-centre observational study. United States | 61 patients who had completed a Whipple procedure | - Prolonged duration of surgery - Type of surgery | SSI |
| Hester *et al* 2015^15^ | Retrospective, single-centre observational study. United Kingdom | 36 revision arthroplasty patients (9 hip, 27 knee) | - Type of surgery - Use of metal implants - The majority of patients had co-morbidities | Overall wound complications |
| Hickson *et al* 2015^16^ | Retrospective before-after, single-centre, observational study. United States | 1948 elective and emergency caesarean section patients | - High risk patients identified (various criteria used) | SSI |
| Holt and Murphy 2015^17^ | Retrospective, single centre, observational study. United Kingdom | 24 oncoplastic breast surgery patients (bilateral surgery) | - The majority of patients had a raised BMI and a large mass of tissue resected | Dehiscence, delayed healing, reoperation rate |
| Matsumoto and Parekh 2015^22^ | Retrospective, before-after, single centre, observational study. United States | 74 total ankle arthroplasty patients | - The majority of patients had a raised BMI and co-morbidities - The use of metal implants | SSI |
| Pellino *et al* 2014a^26^ | Prospective, non-randomised observational study. Italy | 100 patients (50 undergoing breast surgery, 50 colorectal surgery) | - Type of surgery - Prolonged duration of surgery | SSI, length of hospital stay, seroma |
| Pellino *et al* 2014b^25^ | Prospective, non-randomised, pilot, single centre. Italy | 30 Crohn’s disease patients undergoing small bowel resection | - Type of surgery - The majority of patients had co-morbidities and raised ASA score | SSI, length of hospital stay, seroma, reoperation rate |
| Selvaggi *et al* 2014^27^ | Prospective, single centre observational study. Italy | 50 Crohn’s disease patients undergoing abdominal surgery | - Type of surgery - The majority of patients had co-morbidities | SSI, length of hospital stay, readmission rate, reoperation rate, seroma |
| Tan *et al* 2017^29^ | Retrospective, single-centre observational study. Singapore | 42 patients undergoing lower limb bypass surgery | - The majority of patients had co-morbidities and had an emergency procedure - The mean age of participants was >65 years old | SSI, length of hospital stay, readmission rate, reoperation rate |
| van der Valk *et al* 2017^33^ | Prospective, single centre, before-after observational pilot study. Netherlands | 20 patients undergoing abdominoperineal resection for rectal cancer | - The majority of patients had co-morbidities - The mean age of participants was >65 years old - Type of surgery | SSI, dehiscence, reoperation rate, readmission rate, time to healing |
| Conference abstracts | | | |  |
| Hackney and McCoubrey 2017^14^ | Retrospective, single centre observational study. United Kingdom | 71 open abdominal surgery patients | - Type of surgery | Length of hospital stay, readmission rates |
| Irwin *et al* 2018^19^ | Prospective, single centre database audit study. United Kingdom | 155 patients undergoing prepectoral implant-based reconstruction procedures | - The use of metal implants | Dehiscence |
| Kawakita *et al* 2018^21^ | Retrospective, single centre observational study. United States | 759 caesarean section patients | - Inclusion criterion of a BMI ≥40kg/m^2^ | SSI |
| Tuuli *et al* 2017^31^ | Single centre, pilot, open label RCT. United States | 120 caesarean section patients | - Inclusion criterion of a BMI ≥30kg/m^2^ | SSI, seroma, haematoma |
| Zotes *et al* 2015^35^ | Single centre, open label RCT. Mexico | 20 patients undergoing thoracotomy for empyema | - The majority of patients had at least one risk factor (diabetes, poor nutritional status, steroid therapy, prolonged surgery) | Seroma, dehiscence |
